# Investigation of a pathogenic inversion in *UNC13D* and comprehensive analysis of chromosomal inversions across diverse datasets

**DOI:** 10.1038/s41431-025-01817-w

**Published:** 2025-02-28

**Authors:** Tugce Bozkurt-Yozgatli, Ming Yin Lun, Jesse D. Bengtsson, Ugur Sezerman, Ivan K. Chinn, Zeynep Coban-Akdemir, Claudia M. B. Carvalho

**Affiliations:** 1 Department of Biostatistics and Bioinformatics, Institute of Health Sciences, Acibadem Mehmet Ali Aydinlar University, Istanbul, Turkey; 2 Human Genetics Center, Department of Epidemiology, Human Genetics, and Environmental Sciences, School of Public Health, University of Texas Health Science Center at Houston, Houston, TX USA; 3 Pacific Northwest Research Institute, Seattle, WA USA; 4 Department of Biostatistics and Medical Informatics, School of Medicine, Acibadem Mehmet Ali Aydinlar University, Istanbul, Turkey; 5 Department of Pediatrics, Division of Immunology, Allergy, and Retrovirology, Baylor College of Medicine and Texas Children’s Hospital, Houston, TX USA; 6 Center for Human Immunobiology of Texas Children’s Hospital/Department of Pediatrics, Baylor College of Medicine, Houston, TX USA

**Keywords:** Medical genomics, Disease genetics

## Abstract

Inversions are known contributors to the pathogenesis of genetic diseases. Identifying inversions poses significant challenges, making it one of the most demanding structural variants (SVs) to detect and interpret. Recent advancements in sequencing technologies and the development of publicly available SV datasets have substantially enhanced our capability to explore inversions. However, a cross-comparison in those datasets remains unexplored. In this study, we reported a proband with familial hemophagocytic lymphohistiocytosis type-3 carrying a splicing variant (c.1389+1G>A) in trans with an inversion present in 0.006345% of individuals in gnomAD (v4.0) that disrupts *UNC13D*. Based on this result, we investigate the features of potentially pathogenic inversions in gnomAD which revealed 98.9% of them are rare and disrupt 5% of protein-coding genes associated with a phenotype in OMIM. We then conducted a comparative analysis of additional public datasets, including DGV, 1KGP, and two recent studies from the Human Genome Structural Variation Consortium which revealed common and dataset-specific inversion characteristics suggesting methodology detection biases. Next, we investigated the genetic features of inversions disrupting the protein-coding genes. Notably, we found that the majority of protein-coding genes in OMIM disrupted by inversions are associated with autosomal recessive phenotypes supporting the hypothesis that inversions in trans with other variants are potential hidden causes of monogenic diseases. This effort aims to fill the gap in our understanding of the molecular characteristics of inversions with low frequency in the population and highlight the importance of identifying them in rare disease studies.

## Introduction

Inversions are defined as a type of structural variant (SV) that refers to orientation changes in DNA segments. They can be copy-number neutral (classical/simple/balanced) with two breakpoint junctions or be part of complex genomic rearrangements (CGRs) with other copy-number variations (CNVs) [1]. The main mechanism for the formation of classical inversions has been proposed to be non-allelic homologous recombination (NAHR) between inverted repeats [2–4]. Other biological mechanisms may result in inversion formation, including DNA repair-associated events (non-homologous end joining (NHEJ), and microhomology-mediated end joining (MMEJ)) and DNA replication-associated events (e.g., microhomology-mediated break-induced replication (MMBIR)) [1, 5].

Inversions may have an impact on disease phenotypes, often by directly disrupting genes [6]. They may occur within a gene leading to splicing alterations. Mor-Shaked et al. reported a pathogenic inversion in *PRKN*, leading to the skipping of exon 5 in individuals with early-onset Parkinson’s disease (PARK2, OMIM #600116) [7]. Besides, one of the inversion breakpoints may disrupt a gene and result in a disease phenotype [8, 9]. For instance, one of the breakpoints of a 253-kb inversion mapping to intron 30 of *UNC13D* contributes to the manifestation of familial hemophagocytic lymphohistiocytosis 3 (FHL3, OMIM #60898) [10, 11]. In addition to Mendelian disorders, inversions are recognized as significant contributors to common complex disease traits [12–14] and disease prognosis [15]. Additionally, they can also play a role as genetic modifiers affecting disease phenotypes [16]. Moreover, some inversions have no direct effect on disease phenotype by themselves, but they may predispose the loci to further genomic rearrangements with pathogenic consequences [2, 17] including the formation of recombinant chromosomes [1].

Inversion detection is challenging due to their balanced nature and the fact that breakpoints often map to repeats. Those features make them undetectable by comparative genomic hybridization (aCGH) and exome sequencing (ES) [18]. Although short-read whole genome sequencing (WGS) enables the detection of some inversions, it also introduces the issues of false positives and the inability to sequence breakpoint junctions in the repetitive parts of the genome [19, 20]. Long-read WGS technologies, including Pacific Biosciences (PacBio) and Oxford Nanopore (ONT), single-cell template strand sequencing (Strand-seq) [21], and optical genome mapping [22] have improved our ability to detect inversions since these methodologies are more suitable to detect changes within complex repeat regions [4, 23].

Published population datasets using different sequencing technologies like those in Ebert et al. [23], and Porubsky et al. [4], and publicly available databases such as Genome Aggregation Database (gnomAD) [24], The Database of Genomic Variants (DGV) [25], and 1000 Genomes Project (1KGP) [26] provide valuable resources for SV analysis. The recent release of gnomAD dataset version 4 (v4.0) includes short-read genome sequencing data from 63,046 unrelated human samples across the world [24]. The DGV dataset is derived from different methodologies such as sequencing, aCGH, and Fluorescence in situ hybridization (FISH) [25]. Byrska-Bishop et al. released an expanded short-read WGS of 1KGP consisting of 3202 samples, including 602 trios across diverse global populations [26]. Porubsky et al. [4] reported inversions from 41 human samples by integrating Strand-seq [21], haplotype-resolved de novo sequence assemblies generated from PacBio long-reads, and Bionano genomics single-molecule optical mapping [22]. Ebert et al. published 64 assembled haplotypes from 32 diverse human genomes using long-read WGS and strand-seq [23].

**Fig. 1:**
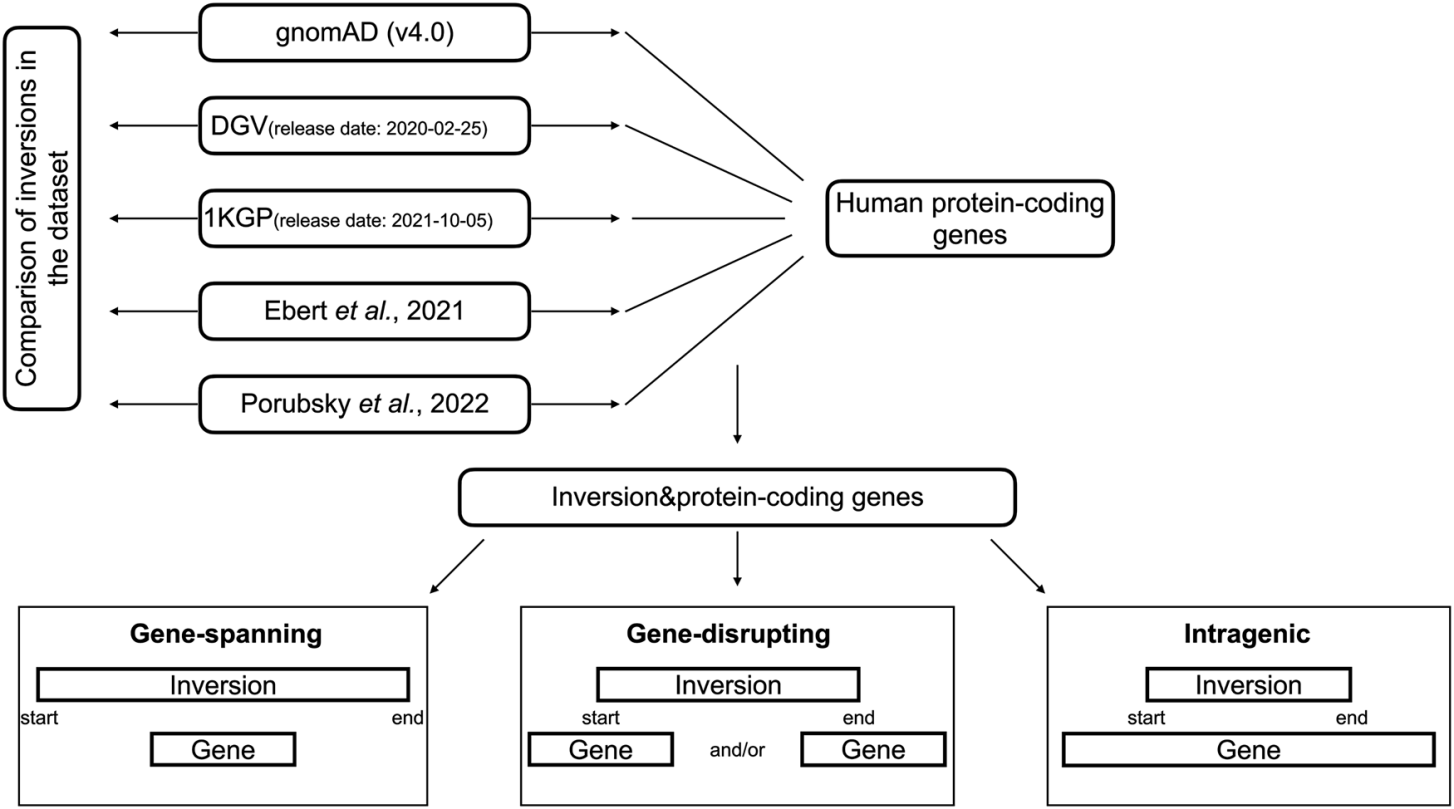
Overview of the datasets and the study design. We extracted inversions from publicly available databases, gnomAD (v4.0) [24], DGV (release date: 2020-02-25) [25], 1KGP (release date: 2021-10-05) [26] and two recent publications of Ebert et al. [23] and Porubsky et al. [4] We then intersect inversions with OMIM genes and grouped inversion-gene intersections into three categories.

Here, we report a proband carrying a pathogenic inversion in trans with a single-nucleotide variant (SNV) affecting *UNC13D*. Then, we comprehensively compare inversions disrupting genes reported in various datasets, gnomAD (v4.0) [24], DGV (release date: 2020-02-25) [25], 1KGP (release date: 2021-10-05) [26], inversions released by Ebert et al. [23] and Porubsky et al. [4] (Fig. 1). Our goal is to provide insights into the features of inversions present in population datasets to genomic disorders.

## Methods

### Case presentation

The proband (SEA110) is a Caucasian white non-Hispanic, non-Latino male born at 31 weeks’ gestation age. Precise parental ancestry remains unknown because parental custody was removed at an early age, and the patient was placed into foster care. He was diagnosed with VACTERL (vertebral defects, anal atresia, cardiac defects, tracheoesophageal fistula, renal anomalies, and limb abnormalities) after birth due to issues including tethered cord and sacral anomalies (requiring cord release), high imperforate anus with rectourethral fistula (treated with diverting loop sigmoidostomy), bronchopulmonary dysplasia with history of pulmonary hypertension, atrioseptal defect, horseshoe kidney, penile hypospadias, diaphragmatic eventration (status post plication), right inguinal hernia (repaired), and G-tube dependence. He did not meet early developmental milestones on time. Other features included microcephaly with plagiocephaly. Type 1 laryngeal cleft was diagnosed at 11 months of age. He also developed kidney stones and hydronephrosis. The patient failed a hearing screen in the left ear. He was also found to have amblyopia and astigmatism and required glasses. The patient had frequent respiratory infections that required supplemental oxygen, including respiratory syncytial virus infection. He presented with pancytopenia at 12 months of life, which was initially felt to be likely viral-mediated. He was hospitalized and discharged. He seemed well but then developed daily fevers and increased stool output. He was re-hospitalized and found to have hepatosplenomegaly by abdominal ultrasound. He then developed acute respiratory failure and required intubation with pressor support. Laboratory testing ultimately confirmed a diagnosis of hemophagocytic lymphohistiocytosis (HLH) by HLH-2004 criteria [27]: fever, splenomegaly, anemia with thrombocytopenia, hypofibrinogenemia, hypertriglyceridemia, hyperferritinemia, elevated soluble interleukin-2 receptor levels, and impaired CD107A mobilization. Initial genetic testing in the first 12 months of life consisted of proband ES and chromosomal microarray testing, both of which were performed by a commercial clinical laboratory. Results were reported as negative for both tests. Upon re-hospitalization, clinical targeted gene panel testing was ordered for inborn errors of immunity and cytopenias, which identified a pathogenic variant at *UNC13D* c.1389+1G>A. As a result of clinical targeted gene panel findings, SEA110 was tested by the Baylor Genetics Clinical Diagnostic Laboratory using rapid short-read WGS. Variant calling used the Illumina Dragen BioIT Platform and detected two heterozygous variants: *UNC13D* c.1389+1G>A and a 253 kb inversion extending from downstream of *LLGL2* (NM_001031803) to exons 31-32 of *UNC13D*.

### Patient sample collection and DNA extraction

Informed consent was obtained for research participation under Pacific Northwest Research Institute approved WCG IRB Protocol #H-47127_20202158.

DNA was extracted from whole blood using the QIAGEN Puregen DNAeasy kit following the manufacturer’s direction with modification of the centrifugation steps, which were extended to 10 minutes. Ultrahigh molecular weight DNA was extracted from whole blood with the Bionano SP-G2 Blood and Cell Culture DNA Isolation Kit (#80060) following the manufacturer’s direction.

### ONT-library preparation and sequencing run

DNA from SEA110 was sheared to an N50 of approximately 10 kb using a Covaris g-TUBE and an Eppendorf 5424 rotor at 5000 rpm. End repair and ligation of adapters for Oxford nanopore sequencing followed the manufacturer’s direction for kit LSK114. Sequencing used Minknow version 23.07.12, with adaptive sampling to enrich for the region of interest. The enrichment region (chr17:75526717-75896404, GRCh38) and reference as a minimap2 index file were provided [28]. Following sequencing, passed reads were re-called using guppy 6.0.1 and the super high accuracy model. Passed reads were mapped to GRCh38 using minimap2 (-Y –secondary=no -a -x map-ont). After mapping, SNVs were called using Clair3 [29] and reads were haplotagged by Whatshap [30].

### Breakpoint junction amplification and Sanger sequencing

Inversion junctions were amplified using primers reported previously with one additional sequencing primer (Supplementary Table 1) [10]. Amplification used the Q5 Polymerase (NEB), and PCR products were gel extracted with the Monarch DNA Gel Extraction kit (NEB) following the manufacturer’s direction. Purified products were sent for Sanger sequencing by GENEWIZ. Sanger sequencing was analyzed using Geneious Prime software (Dotmatics).

### Optical genome mapping

Ultrahigh molecular weight DNA (UHMW) was labeled with the Bionano Direct Label and Stain-G2 (DLS2-G2) Kit (#80046) following the manufacturer’s direction. In brief, 750 ng of UHMW DNA was labeled with a proprietary green fluorophore (DL-Green), and after purification, the DNA backbone was stained with a proprietary DNA stain. After staining, the sample was run on a Bionano Saphyr instrument. A de novo assembly was generated in Bionano access version 1.8.1, with a molecule N50 of 150.38 kb in length and 15.61 labels per 100 kb. The resulting assembly was compared to the hg38 reference genome, variants were called using Bionano solve version 1.8.1.

### Datasets utilized in this study

We analyzed the inversions mapped to the reference human genome of hg38 from three publicly accessible databases, gnomAD (v4.0) [24], DGV (release date: 2020-02-25) [25] and 1KGP (release date: 2021-10-05) [26], and two recent studies of Ebert et al. [23] and Porubsky et al. [4] (Fig. 1). We extracted inversion calls in autosome (chr1-22) and sex (chrX and chrY) chromosomes from the datasets. The gnomAD (v4.0) [24] SV dataset was downloaded from https://gnomad.broadinstitute.org/downloads. The DGV [25] SV dataset was downloaded from the link: http://dgv.tcag.ca/dgv/docs/GRCh38_hg38_variants_2020-02-25.txt. DGV [25] includes inversions from several studies (Supplementary Table 2) derived from different methodologies, including sequencing, oligo aCGH, and FISH. We included inversions detected by all of these studies. SV data in the 1KGP was downloaded from the following link: https://www.internationalgenome.org/data-portal/data-collection/30x-grch38. The updated callset to the original release of the inversions reported by Ebert et al. [23] was downloaded from the following link: http://ftp.1000genomes.ebi.ac.uk/vol1/ftp/data_collections/HGSVC2/release/v2.0/integrated_callset/. Lastly, we included the inversions reported by Porubsky et al. [4].

### Gene annotations

We downloaded the gene regions with their canonical transcripts present in the hg38 version of the GENCODE (v46) database (Data update date: 2024-04-02) through the University of California Santa Cruz (UCSC) [31] to identify the inversions intersecting with the human protein-coding genes. We filtered the dataset to extract only the genes with protein-coding transcripts, excluding those with other transcript types. (Supplementary Fig. 1). Then, we retained the genes in human autosome chromosomes (chr1-22) and sex chromosomes (chrX and chrY). We also downloaded the dataset of the Online Mendelian Inheritance in Man (OMIM) (data freeze date: 06-18-2024) [32] (https://www.omim.org/downloads) as well as rare disease-related genes in Orphanet data (https://www.orphadata.com/genes/).

### Analysis of inversions across datasets and protein-coding genes

We used the Bedtools (v2.30.0) [33] intersect function with the fraction option 0.5 to detect the overlap between inversion locations in different datasets. Bedtools intersect function takes a genomic feature as the first input and finds overlapped regions between another genomic feature as the second input. The fraction option 0.5 allows us to find the overlap, including at least 50% of the sequence length of inversions. We also implemented the Bedtools (v2.30.0) [33] intersect function with the default parameters to detect the overlap between inversions and protein-coding genes. The intersections between inversions and human protein-coding genes were classified into three distinct categories. In the first category (Gene-spanning), inversion breakpoints do not map to genes; in the second category (Gene-disrupting), at least one of the inversion breakpoints maps within a gene; in the third category (Intragenic), both inversion breakpoints map within a single gene (Fig. 1). Notably, majority of intragenic inversions affect a single intron (1356/1586) and tend to occur in larger introns (Supplementary Fig. 2A and B). Strikingly, the mean size of canonical introns intersecting with intragenic inversions are significantly larger compared to the mean size of all intronic regions of protein-coding genes (*p*-value < 2.2e−16, Wilcoxon test, Supplementary Fig. 2B and C). We included those types of inversion since they still have the potential of disrupt critical regulatory elements or functional motifs that significantly impact gene expression and function or alter splicing [34].

### Enrichment analysis of the genes intersecting inversions

We performed gene set enrichment analysis with the protein-coding genes overlapping with inversions in categories of gene-disrupting and intragenic by applying Enrichr [35]. The list of the genes intersecting inversions in each intersection category was given as input to Enrichr [35]. Then, we reported the Human Phenotype Ontology (HPO) terms enriched by these genes.

### Prediction of long-range effect of gene-spanning inversions

We applied POSTRE [36] to the gene-spanning inversions intersecting protein-coding genes. We convert the inversion positions from hg38 to hg19 using the UCSC liftOver tool (https://genome.ucsc.edu/cgi-bin/hgLiftOver) since POSTRE [36] accepts SV data in hg19 with some predefined phenotype information (i.e. cardiovascular, head&neck, limbs, neurodevelopmental, vision&eye).

### Computational analysis

Computational analyses were carried out using R (v.4.2.0) [37]. The plots were generated using the package ggplot2 [38] and the UpSet R package [39].

## Results

### A pathogenic *UNC13D* inversion is present in gnomAD

**Fig. 2:**
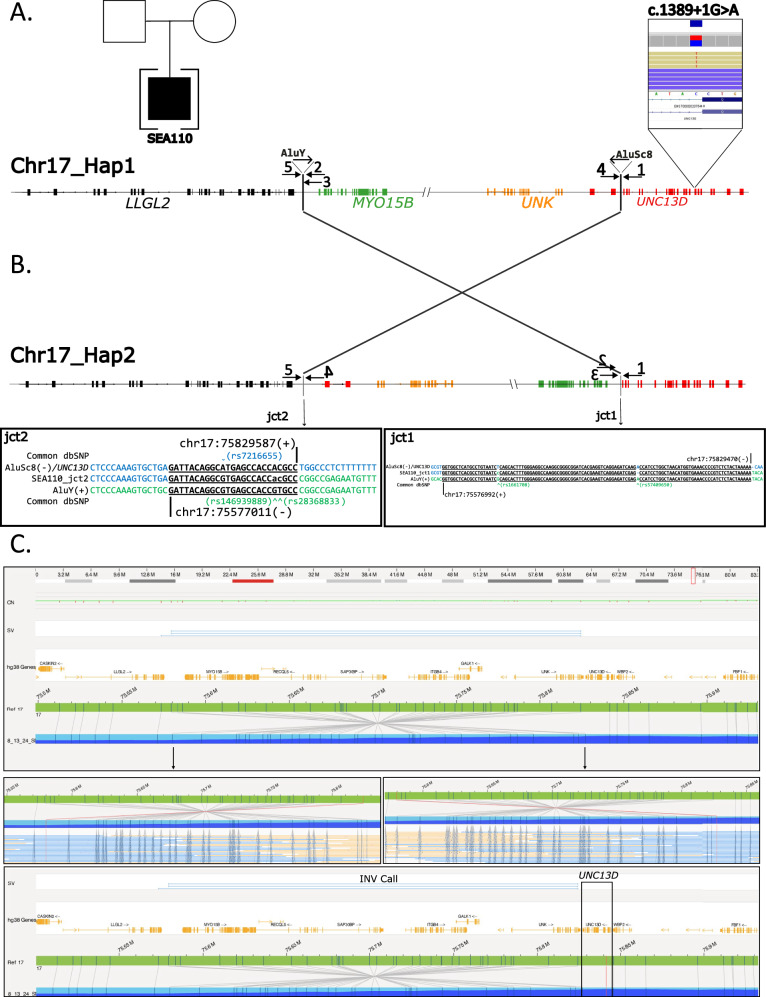
*UNC13D* variants detected in a proband diagnosed with FHL3. **A** Pedigree of patient SEA110 and IGV screenshot displaying nanopore sequencing reads that detected the pathogenic SNV in *UNC13D* (NM_199242.3). Chr17_Hap1 represents the haplotype carrying the SNV in *UNC13D*, the blowout of *UNC13D* points to the approximate location of the SNV. **B** Diagram of Chr17_Hap2, showing the inversion junction sequencing alignments of each breakpoint. Arrows point to the alignments for junctions 1 and 2 (jct1/2). PCR primers used to obtain the breakpoint junctions for Sanger sequencing are indicated by arrows. Arrows are not to scale. **C** Optical Genome Mapping showing the inversion in Chr17_Hap2, panels show molecules spanning each junction and the location of *UNC13D* relative to the inversion call.

We identified an inversion accompanied by the canonical donor splice site SNV in *UNC13D* in SEA110 (Fig. 2 and Supplementary Fig. 3). The 253-kb inversion has been documented in individuals with Swedish ancestry and reported to cause FHL3 when inherited as homozygous or in trans with pathogenic SNVs and small indels in *UNC13D* [10, 11]. We observed an almost identical inversion reported in gnomAD at coordinates chr17:75576924-75829482 (INV_CHR17_66182818), which is present in 0.006345%, exclusively in heterozygous state in individuals from European Finnish and Admixed American populations (Supplementary fig. 4). The SEA110 inversion shows two breakpoint junctions with 111 (junction 1) and 23 (junction 2) nucleotides similarity generated by *Alu*-*Alu* mediated rearrangement (AAMR) (Fig. 2). Parental samples are not available to test for inheritance; therefore, we do not have information about ancestry and cannot investigate whether this inversion is the same reported in gnomAD (a potential founder event) or if it is a recurrent inversion generated independently via AAMR in this proband. Optical Genome Mapping supports the breakpoint junctions of the inversion obtained by Sanger sequencing. The detected inversion has multiple molecules spanning both breakpoints and several molecules spanning the entire inversion supporting the inversion call. Bionano solve software called the inversion as heterozygous, but lack of label density in *UNC13D* results in the exclusion of *UNC13D* from the called inversion. ONT sequencing was applied to confirm heterozygosity, and manual phasing indicated the pathogenic SNV and inversions are in trans (Supplementary Fig. 3B).

### Inversions in gnomAD (v4.0) are rare and affect protein-coding genes

We hypothesized that pathogenic inversions are present as rare alleles in the general population. To investigate this concept, we categorized 2185 inversions in gnomAD into two groups: Rare (allele frequency <5%) and common (allele frequency ≥5%). Altogether, 2,161 (98.9%) inversions are rare; 24 inversions (1.1%) are common in gnomAD (Supplementary Fig. 5A).

We investigated the human protein-coding genes affected by rare and common inversions in gnomAD. We analyzed 19,697 protein-coding genes in GENCODE (v46); 4921 are related to a phenotype in OMIM, 11,306 are not yet linked with a phenotype in OMIM, and 3470 genes are not cataloged in OMIM. We overlapped inversions in gnomAD and protein-coding genes and categorized the intersections into three groups (gene-spanning, gene-disrupting, and intragenic). Next, we focused on the inversions in categories of gene-disrupting and intragenic since they can be critical mechanisms for disease pathology (Supplementary Table 3). 279 rare gnomAD inversions affect 5% of genes associated with a phenotype in OMIM (247 out of 4,921; Supplementary Fig. 5C) in contrast with 4.6% of genes not associated with a phenotype in OMIM (521 out of 11,306; Supplementary Fig. 5C) based on categories of gene-disrupting and intragenic. Furthermore, 254 out of 279 rare gnomAD inversions have not been found in the homozygous state and affect 106 autosomal recessive (AR) disease genes (Supplementary Table 4).

### Features of the inversions reported in distinct datasets

To compare the characteristics of inversions in gnomAD [24] with other publicly available datasets, we conducted a comparative analysis using inversion data from DGV [25], 1KGP [26], and two recent publications of Ebert et al. [23] and Porubsky et al. [4] (Fig. 1).

We extracted 2185 inversions from gnomAD, 3468 inversions from DGV, 920 inversions from 1KGP, 414 inversions from the data released by Ebert et al., and 339 inversions from the call set published by Porubsky et al. The summary statistics of inversion length in each dataset are provided in Table 1. gnomAD shows a more even distribution regarding size and displays the largest events (Supplementary Fig. 6), including a 118.67 Mb pericentric inversion (INV_CHR5_77480914). Most of DGV inversions (75%) are between 0.035 kb and 24.22 kb. 1KGP inversions tend to be smaller as the median length of 0.831 kb, whereas Ebert et al. and Porubsky et al. show the highest median length of 293.19 kb and 251.71 kb, respectively.

**Table 1:** Summary statistics of the datasets analyzed in this study.

Dataset	Sequencing technology	Number of inversions	Minimum length (kb)	1st Quartile (kb)	Median length (kb)	Mean length (kb)	3rd Quartile (kb)	Maximum length (kb)
gnomAD-SV (v4.0)	Short-read WGS	2185	0.052	0.896	7.1	2402.4	323.63	118,667.16
DGV (release date: 2020-02-25)	Mixed	3468	0.035	0.395	2.67	168.5	24.3	9734
1KGP (release date: 2021-10-05)	Short-read WGS	920	0.052	0.238	0.831	9.41	6.16	98.73
Ebert et al. [23]	Long-read WGS, Strand-seq	414	0.3	8.13	23.94	293.19	87.63	57,207.41
Porubsky et al. [4]	Long-read WGS, Strand-seq, Single-molecule optical mapping	399	0.236	4.67	20.73	251.71	114.28	23,268.23

### Estimating redundancy among the inversions available from different datasets

We investigated the number of common and dataset-specific inversions across different datasets using very stringent criteria based on the start and end locations of the inversions (Supplementary Fig. 7). Redundancies in the datasets are expected due to the overlap of samples reported in distinct publications (e.g., Ebert et al. and Porubsky et al.*)* or inclusion of datasets into publicly shared ones (e.g., gnomAD v.2 is included in DGV). We observed very little redundancy for inversions among the individual datasets (Supplementary Fig. 7) because the different applied sequencing technologies provided distinct resolutions concerning breakpoint junctions. We then decreased the stringency to intersect inversions in each dataset with at least 50% of their sequence (Supplementary Fig. 8). The inversions in gnomAD and DGV share (49.4% and 77.2%) more inversions with each other compared to other datasets. 78.3% of 1KGP inversions overlap with at least one inversion in gnomAD. Around 70% of inversions in Ebert et al. and Porubsky et al. overlap with each other.

### Inversions disrupting genes

**Fig. 3:**
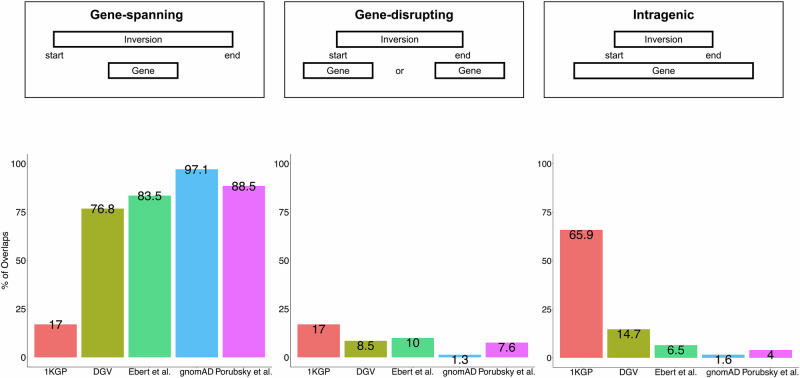
Percentages of inversions in different categories across datasets. We grouped the intersections between inversions and OMIM phenotype-related genes into three categories. The first category comprises genes covered by inversions (gene-spanning), the second category includes intersections where one of the inversion breakpoints is located within a gene region (gene-disrupting), and the third category involves inversions occurring within a gene region (intragenic).

We overlapped the inversions in the datasets with the protein-coding genes. Then, we classified the overlaps between inversions and protein-coding genes into three categories, as defined previously defined in this manuscript (Fig. 1). The majority of the overlaps from all datasets, except 1KGP, map with the category of gene-spanning (76.8% in DGV to 97.1% in gnomAD). 65.9% of inversion-gene intersections belong to the category of intragenic in 1KGP (Fig. 3).

Next, we focused on the inversions in categories of gene-disrupting and intragenic since they can be critical mechanisms for disease pathology (Supplementary Tables 5 and 6). We delved deep into the protein-coding genes associated with clinical phenotypes in OMIM disrupted by inversions in this intersection between categories of gene-disrupting and intragenic. In total, 847 inversions have one breakpoint junction mapping to 830 protein-coding genes based on the category of gene-disrupting and can be potentially relevant to genetic disorders (Supplementary Table 5). On the other hand, in total, breakpoint junctions of 1586 inversions are within 1030 protein-coding genes based on the category of intragenic and can also be potentially relevant to genetic disorders (Supplementary Table 6). Interestingly, both DGV and gnomAD inversions show higher frequencies of disrupting genes associated with disease compared to other datasets (1.6% and 2.1%, respectively) in the category of gene-disrupting (Supplementary Fig. 9A). Importantly, inversions in both datasets also disrupt a higher proportion of OMIM phenotype-related genes in the category of intragenic (3.7% and 3.2%, respectively), while Porubsky et al. has a smaller proportion (0.2%, Supplementary Fig. 9B). The inheritance pattern of the genes overlapping with inversions for categories of gene-disrupting and intragenic for each dataset is given in Supplementary Fig. 9C and D. About 40.9% and 50% of the inversions in both categories of gene-disrupting and intragenic regardless of dataset are in AR disease genes (Supplementary Fig. 9C and D). Autosomal dominant (AD) inheritance is the second most prominent disease gene pattern (16.7% and 33.8%, Supplementary Fig. 9C and D).

We then performed gene set enrichment analysis with all protein-coding genes intersecting with all gene-disrupting and intragenic inversions in the datasets. All enriched HPO terms belonging to each category are provided in Supplementary Tables 7 and 8.

## Discussion

In this study, we reported a case with c.1389+1G>A and NC_000017.11: 75576992_75829587inv in *UNC13D* presenting with an FHL3 phenotype. c.1389+1G>A in the exon 15 splice donor site has been previously reported in several studies and has been shown that it caused exon 14 to be incorrectly spliced to exon 16, skipping exon 15 entirely [40, 41]. The inversion in the patient disrupts *UNC13D* following the category of gene-disrupting (Fig. 2). The pathogenic inversion is present in heterozygosity in gnomAD (v4.0) in individuals from European Finnish and Admixed American populations (Supplementary Fig. 4). To identify other inversions that are likely pathogenic similar to the one affecting *UNC13D*, we delved deep into gnomAD inversions. There are 279 rare inversions in gnomAD affecting 247 protein-coding genes associated with a phenotype in OMIM based on categories of gene-disrupting and intragenic; 254 of them have not been found in the homozygous state and overlap with 106 AR disease genes (Supplementary Table 4), similar to the overlap between INV_CHR17_66182818 and *UNC13D*. For instance, 115,736-bp inversion in gnomAD, INV_chr1_04df2580, (https://gnomad.broadinstitute.org/variant/INV_CHR1_04DF2580?dataset=gnomad_sv_r4) disrupts *DPYD* with the breakpoint junctions in intron 12 and intron 8. Van Kuilenburg et al. has reported a 115,731-bp inversion with breakpoints in intron 8 and intron 12 of *DPYD* in a patient with Dihydropyrimidine dehydrogenase deficiency (OMIM #274270) [42].

Then, we conducted analyses on inversions from diverse datasets. It is important to highlight that these inversions were derived from different sequencing technologies (Table 1). While the inversions in 1KGP and gnomAD were detected using short-read WGS, the inversions reported by Ebert et al. and Porubsky et al. were identified by long-read WGS and Strand-seq. Strand-seq was shown to be the ideal technology to detect inversions, especially those mediated by large segmental duplications or other genomic repeats which often happen as a result of NAHR; 72% of balanced inversions in Porubsky et al. are generated by NAHR [4, 23]. In contrast, short-reads are not suitable to identify such inversions, although it can resolve inversions with blunt or microhomology at the breakpoint junctions such as those generated by NHEJ [1]. Therefore, while we expected to detect redundancy among datasets, we also expected to identify unique inversions only identifiable by certain methodologies but invisible to others. While between 11.1% to 49.4% of the inversions in gnomAD overlap with inversions in other datasets, from 21.6% to 76.4% of inversions in Porubsky et al. overlap with inversions in other datasets. Strikingly, gnomAD (v4.0) has inversions with a longer length and a higher number of larger inversions (median length of 7.1 kb), which raises the question of whether Mb size inversions, including pericentric ones, are more often generated by NHEJ (Table 1). In fact, we have investigated large inversions detected by karyotyping (8 Mb to 178 Mb) in a diagnostic setting and found that none of the resolved inversions (13/18 or 72%) are mediated by repeats [1] which has been confirmed by a second more recent study [43]. Besides, it should be taken into account that these inversions were generated by different SV callers, and these tools exhibit different false positive rates [19, 44]. Of note, the majority of inversions from the datasets included in this study (between 65–86%) were validated by multiple approaches [4, 23, 24, 26]. Also, redundancies in these datasets will occur due to the same ancestral inversions being reported from distinct individuals while identified by distinct technologies, due to analysis of similar samples or due to the incorporation of entire datasets into larger ones, e.g., DGV incorporates 1KGP phase 3 (Supplementary table 2).

Next, we examined whether the inversions in all datasets disrupt human protein-coding genes by classifying inversion-gene intersections into three different categories (Fig. 1). The majority of the overlaps in all datasets except 1KGP are from the category of gene-spanning (Fig. 3) which is consistent with the small inversion sizes in 1KGP (Supplementary Fig. 6, Table 1). These results also highlight the fact that inversions in 1KGP often have both breakpoints within the same gene which potentially can lead to truncated transcripts subjected to nonsense mediated decay (NMD) or to exon skipping. In contrast, 97.1% of intersections in gnomAD belong to the category of gene-spanning, consistent with gnomAD presenting longer inversions compared to other datasets (Table 1). We focused most of the analysis on categories of gene-disrupting and intragenic inversions which may have an impact on protein-coding genes and cause diseases most likely by loss-of-function effects similarly to disruption of *UNC13D*. However, it is important to highlight that gene-spanning inversions may have a long-range effect on disease pathomechanisms. In fact, based on the POSTRE [36] prediction tool, about 14% of gene-spanning inversions are predicted to have a potential long-range effect (Supplementary Table 9).

**Fig. 4:**
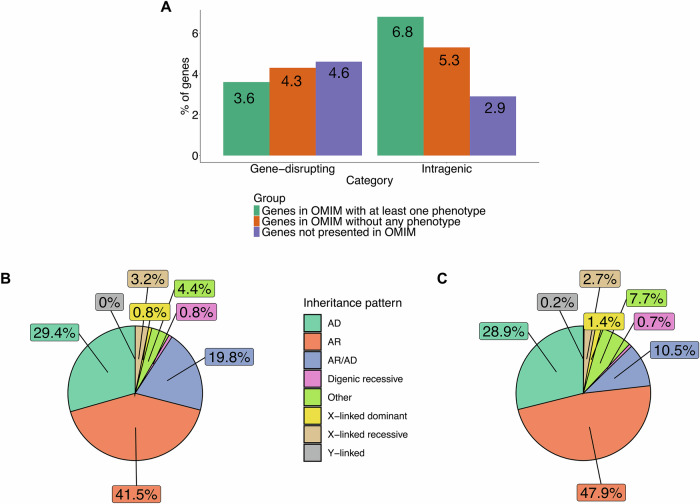
Features of protein-coding genes overlapping with gene-disrupting and intragenic inversions. **A** Percentage of protein-coding genes overlapping with the gene-disrupting and intragenic inversions across datasets. **B** Inheritance pattern of the genes overlapping with all gene-disrupting inversions. **C** Inheritance pattern of the genes overlapping with all intragenic inversions.

Next, we focused on the protein-coding genes that are associated with a phenotype in OMIM disrupted by inversions. Upon examining the genes overlapping with the inversions in categories of gene-disrupting and intragenic, we found that most genes intersecting with inversions across all datasets belong to the AR group, while AD disease genes are the second most prominent group. (Fig. 4B and C). While the observed 41.5% of autosomal recessive genes in the category of gene-disrupting could be a random occurrence that reflects the distribution in OMIM (Supplementary Fig. 10, Binomial test, *p-*value = 0.797), the proportion in the category of intragenic (47.9%) is significantly different from the OMIM distribution (Supplementary Fig. 10, Binomial test, *p-*value = 4.7085e−37). Inversions that disrupt AD disease genes can also be particularly noteworthy, as they might introduce genomic instability in these regions, potentially leading to the formation of other SVs [17].

The number of inversions involving protein-coding genes associated with one or more phenotypes is markedly distinct in each dataset, with gnomAD and DGV showing a higher overlap rate with OMIM phenotype-related genes than other datasets (Supplementary Fig. 9). We observed that the genes disrupted by inversions in categories of gene-disrupting and intragenic are associated with both Mendelian disorders, such as Spinocerebellar ataxia 31 (OMIM #619422), and complex disease traits, such as susceptibility to autism (OMIM #618830).

We further performed gene set enrichment analysis on the genes interrupted by inversions in categories of gene-disrupting and intragenic. All enriched HPO terms except Autosomal dominant inheritance (HP:0000006) for the category of intragenic are statistically insignificant (Supplementary Tables 7 and 8). This result might be expected since we used diverse genes that overlap inversions in the whole genome. Nevertheless, we still report the list of HPO terms enriched by the genes disrupted by inversions to be able to gain an insight into these genes and their related phenotypes.

Finally, sequencing technologies, including short-read WGS, long-read WGS, Strand-seq, and optical mapping, have significantly contributed to the discovery of inversions. Publicly accessible datasets using these technologies are important resources that may facilitate discoveries of pathogenic inversions underlying various disease traits. This study sheds light on the possible impact of the inversions in these datasets on revealing disease phenotypes.

## Supplementary information

Legends of the supplementary materials

Supplementary Figures

Supplementary Tables

## Data Availability

Gnomad SV data: https://gnomad.broadinstitute.org/downloads. DGV SV data: http://dgv.tcag.ca/dgv/docs/GRCh38_hg38_variants_2020-02-25.txt 1KGP SV data: https://www.internationalgenome.org/data-portal/data-collection/30x-grch38. The updated callset to the original release of the inversions reported by Ebert et al. [23]: http://ftp.1000genomes.ebi.ac.uk/vol1/ftp/data_collections/HGSVC2/release/v2.0/integrated_callset/. GENCODE v46: https://genome.ucsc.edu/cgi-bin/hgTables. OMIM gene list: https://www.omim.org/downloads. Orphanet gene list: https://www.orphadata.com/genes/. Data generated in this study are deposited in the Sequence Read Archive (SRA), accession number SRR31350946.

## Code availability

The script for data analysis in this manuscript is available at https://github.com/Carvalho-Lab/Tugce_INV/tree/main.
