# Supplementary material for: Investigation of a pathogenic inversion in UNC13D and comprehensive analysis of chromosomal inversions across diverse datasets
Source: Eur J Hum Genet. 2025 Feb 28;33(7):887–95. doi: 10.1038/s41431-025-01817-w (PMC12229492; doi:10.1038/s41431-025-01817-w)
Supplement: Supplementary file 1 — Legends of the supplementary materials [file 41431_2025_1817_MOESM1_ESM.docx]

**SUPPLEMENTARY INFORMATION**

Supplementary Figure 1. Barplot of the transcript types represented in GENCODE v46.

Supplementary Figure 2. Boxplots of inversion and intron length across databases. (A) Boxplot of inversion length (log10 transformed) in each category. (B) Boxplot of length (log10 transformed) of intronic regions intersecting with intragenic inversions. (C) Boxplot of intron length (log10 transformed) from canonical transcripts of protein-coding genes.

Supplementary Figure 3. IGV visualization of DNA variants at the inversion locus shown by adaptative sampling nanopore sequencing. (A) Illustration of which mapped reads correspond to each junction, reads mapping to jct2 are the reads highlighted in red with soft clipping extending to green, while reads highlighted in green with soft clipping extending into red map to junction 1. (B) Manual phasing of c.1389+1G>A to the non-inverted haplotype. Black boxes highlight SNPs that are unique to the SNV haplotype. The dashed black line indicates a read that extends past jct2 from the inversion and contains c.1389+1G>A.

Supplementary Figure 4. Allelic frequency information for the *UNC13D* inversion across different ancestries in gnomAD.

Supplementary Figure 5. Rare and common inversions in gnomAD. (A) 99% of inversions in gnomAD v4.0 are rare with <0.5% frequency. (B) Number of genes intersecting with common inversions in gnomAD v4.0 based on categories of gene-disrupting and intragenic. (C) Number of genes intersecting with rare inversions in gnomAD v4.0 based on categories of gene-disrupting and intragenic.

Supplementary Figure 6. Distribution of inversion length across datasets.

Supplementary Figure 7. Upset plot displaying the number of shared inversions (100% overlap with defined start and end coordinates) across datasets.

Supplementary Figure 8. Barplots displaying percentages of shared inversions with at least 50% overlap across each pair of dataset.

Supplementary Figure 9. Distribution and inheritance of protein-coding gene that are related to a phenotype in OMIM overlapping inversions. (A) Percentage of the OMIM phenotype-related genes overlapping inversions in the category of gene-disrupting. (B) Percentage of the OMIM phenotype-related genes overlapping inversions in the category of intragenic. (C) Pie charts of inheritance patterns of genes overlapping with inversions in each dataset based on the category of gene-disrupting. (D) Pie charts of inheritance patterns of genes overlapping with inversions in each dataset based on the category of intragenic.

Supplementary Figure 10. Inheritance pattern of the genes related to a phenotype in OMIM.

Supplementary Table 1. Primer sets used in the study.

Supplementary Table 2. References of DGV inversions.

Supplementary Table 3. Rare gnomAD inversions-genes intersections in categories of gene-disrupting and intragenic.

Supplementary Table 4. Rare gnomAD inversions with homozygous frequency 0 - OMIM AR genes intersections in categories of gene-disrupting and intragenic.

Supplementary Table 5. Inversion-gene intersections from all datasets in the category of gene-disrupting.

Supplementary Table 6. Inversion-gene intersections from all datasets in the category of intragenic.

Supplementary Table 7. Enriched HPO terms for the protein-coding genes intersecting with the inversions in all datasets in the category of gene-disrupting.

Supplementary Table 8. Enriched HPO terms for the protein-coding genes intersecting with the inversions in all datasets in in the category of intragenic.

Supplementary Table 9. Gene-spanning inversions with a predicted long-range effect by POSTRE [36].
