# Supplementary material for: Investigation of a pathogenic inversion in UNC13D and comprehensive analysis of chromosomal inversions across diverse datasets
Source: Eur J Hum Genet. 2025 Feb 28;33(7):887–95. doi: 10.1038/s41431-025-01817-w (PMC12229492; doi:10.1038/s41431-025-01817-w)
Supplement: Supplementary file 2 — Supplementary Figures [file 41431_2025_1817_MOESM2_ESM.pdf]

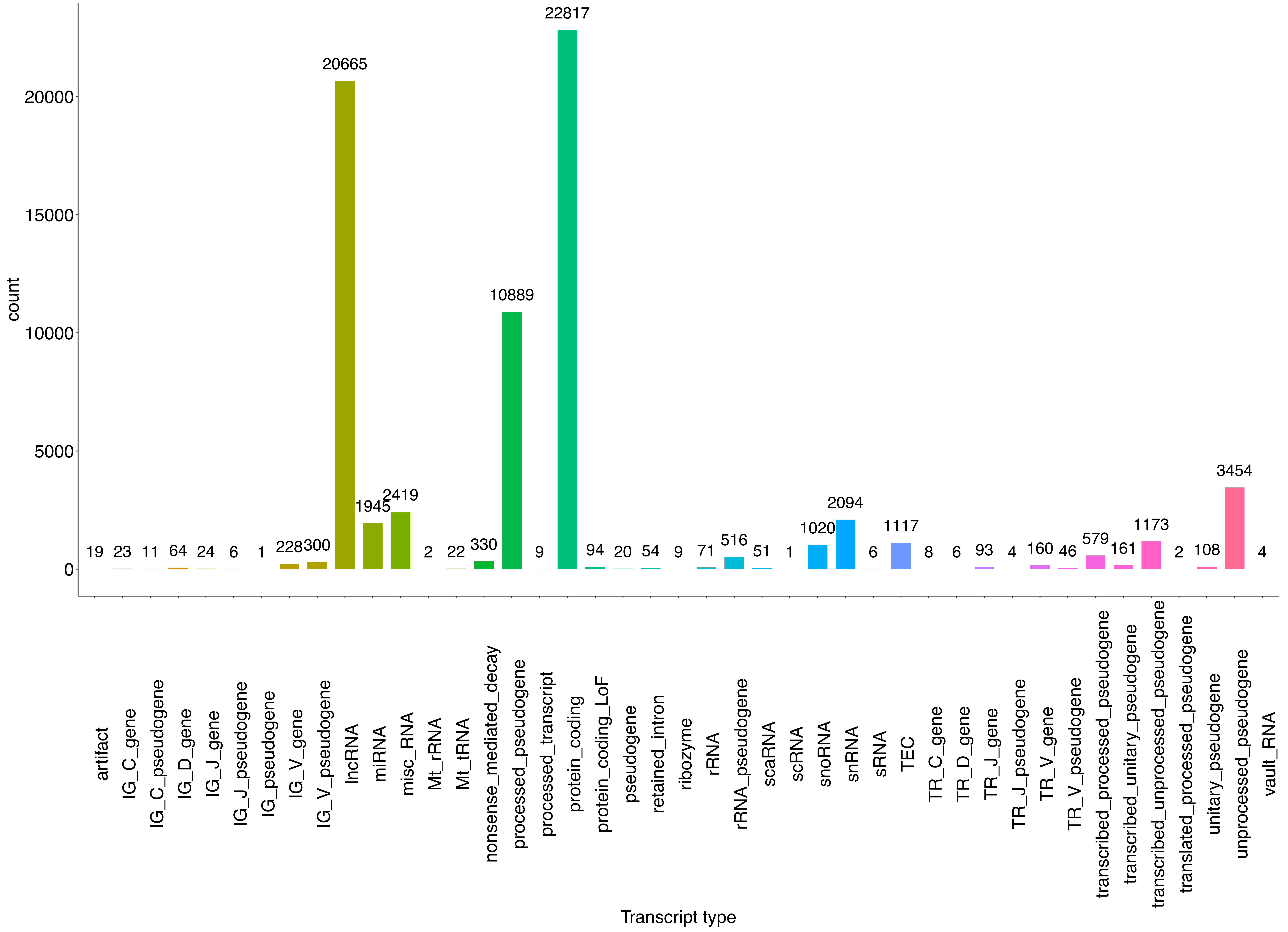

Supplementary figure 1

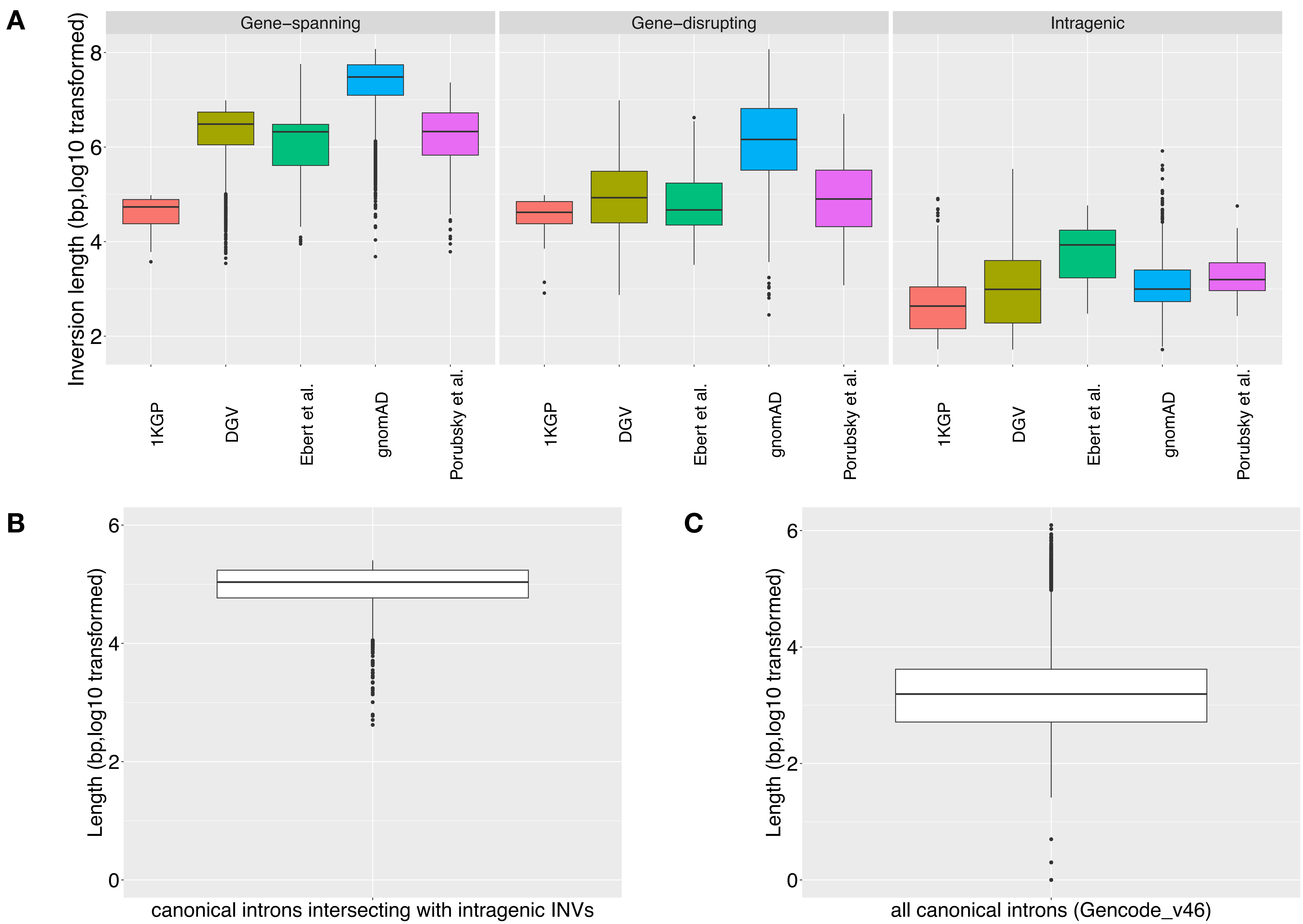

Supplementary figure 2

A

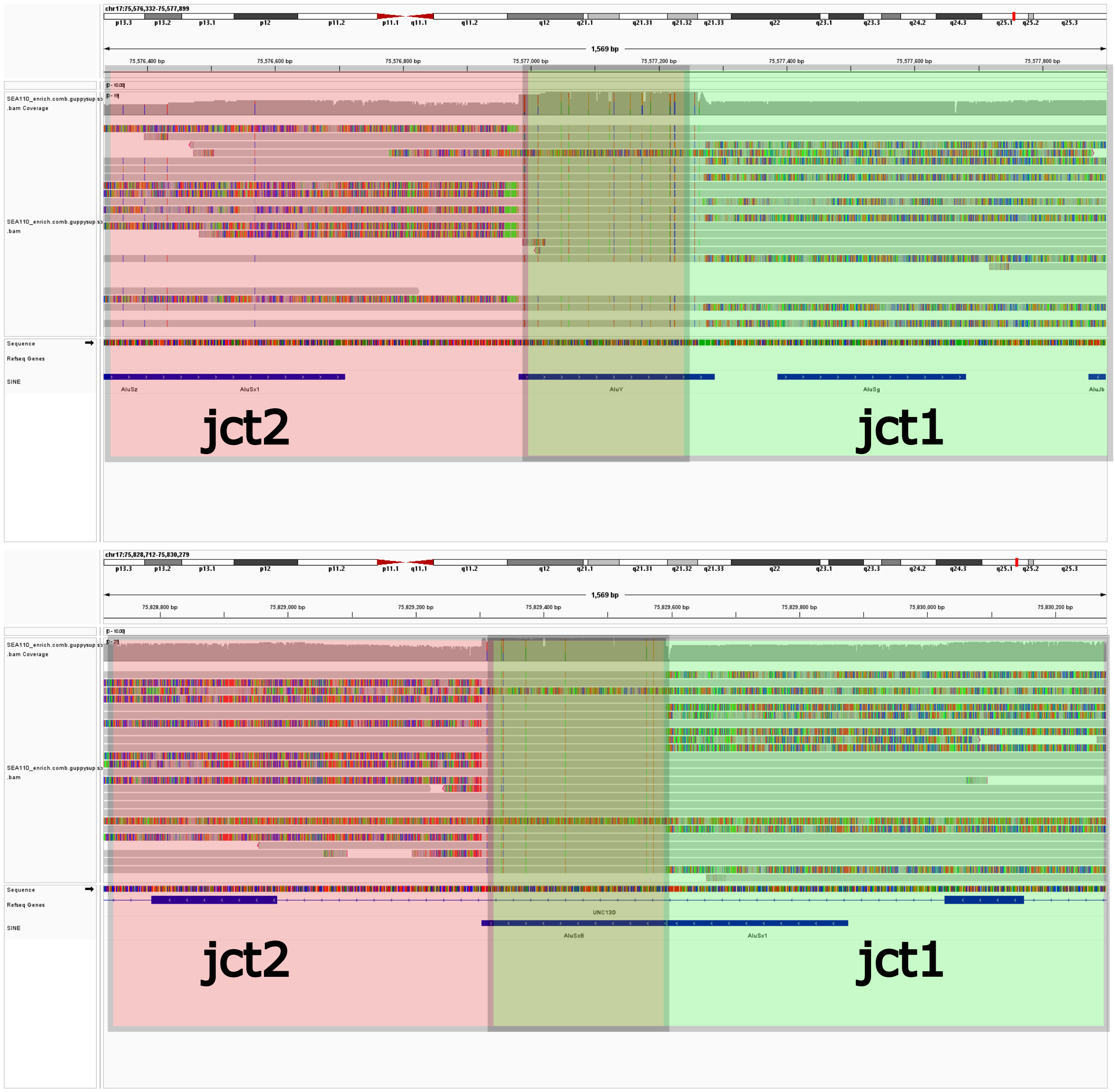

B

SNV (manual phasing)

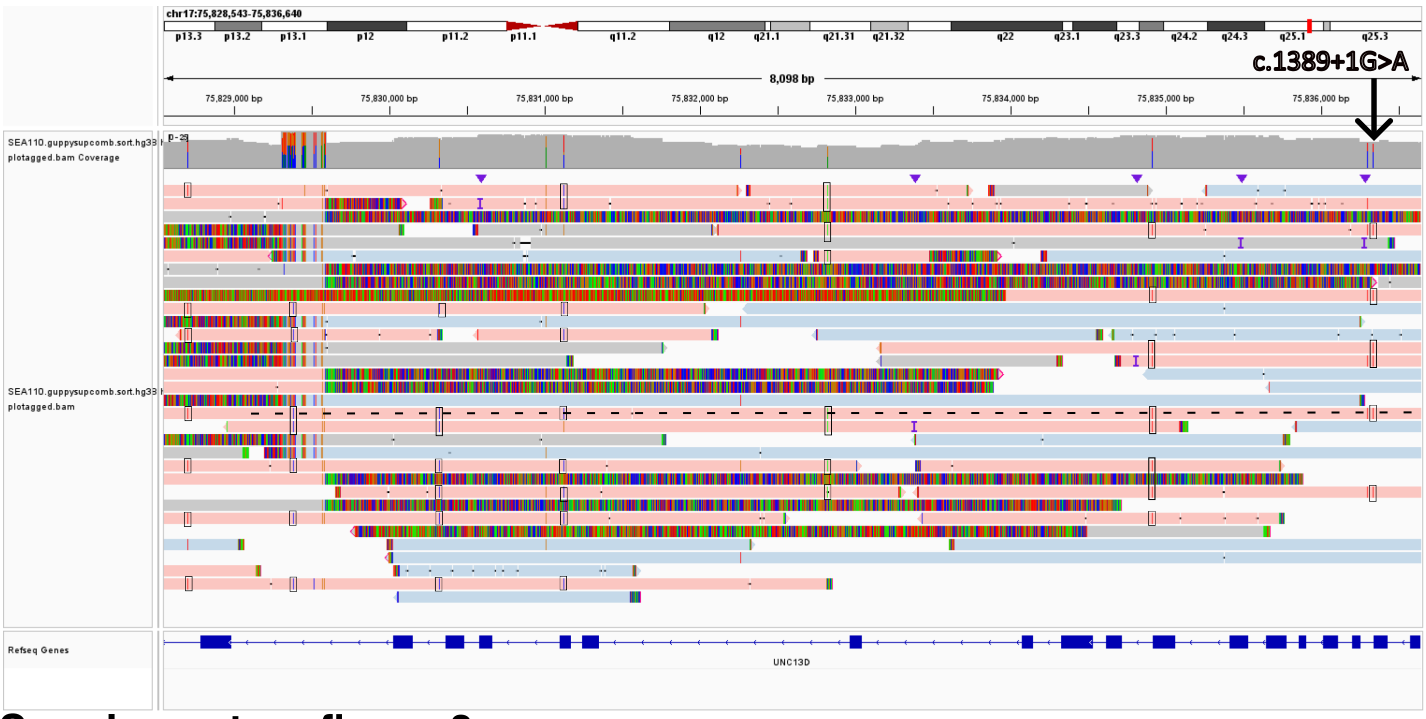

Supplementary figure 3

Filter Pass
Allele Count 8
Allele Number 126092
Allele Frequency 0.00006345
Quality score 1
Position 17:75576924-75829482
Size 252,558 bp
Class inversion
Evidence Anomalous paired-end reads
Algorithms TEXT NEEDED FOR ALGORITHM LABEL "rescan", Manta

External Resources

- UCSC

Feedback

Report an issue with this variant

Genetic Ancestry Group Frequencies

Genetic Ancestry Group	Allele Count	Allele Number	Number of Homozygotes	Allele Frequency
Admixed American	2	12594	0	0.0001588
European (Finnish)	1	6476	0	0.0001544
European (non-Finnish)	5	59088	0	0.00008462
African/African American	0	33816	0	0.000
Amish	0	842	0	0.000
Ashkenazi Jewish	0	3180	0	0.000
East Asian	0	4054	0	0.000
Middle Eastern	0	64	0	0.000
Remaining	0	1572	0	0.000
South Asian	0	4406	0	0.000
XX	3	65800	0	0.00004559
XY	5	60292	0	0.00008293
**Total**	**8**	**126092**	**0**	**0.00006345**

Supplementary figure 4

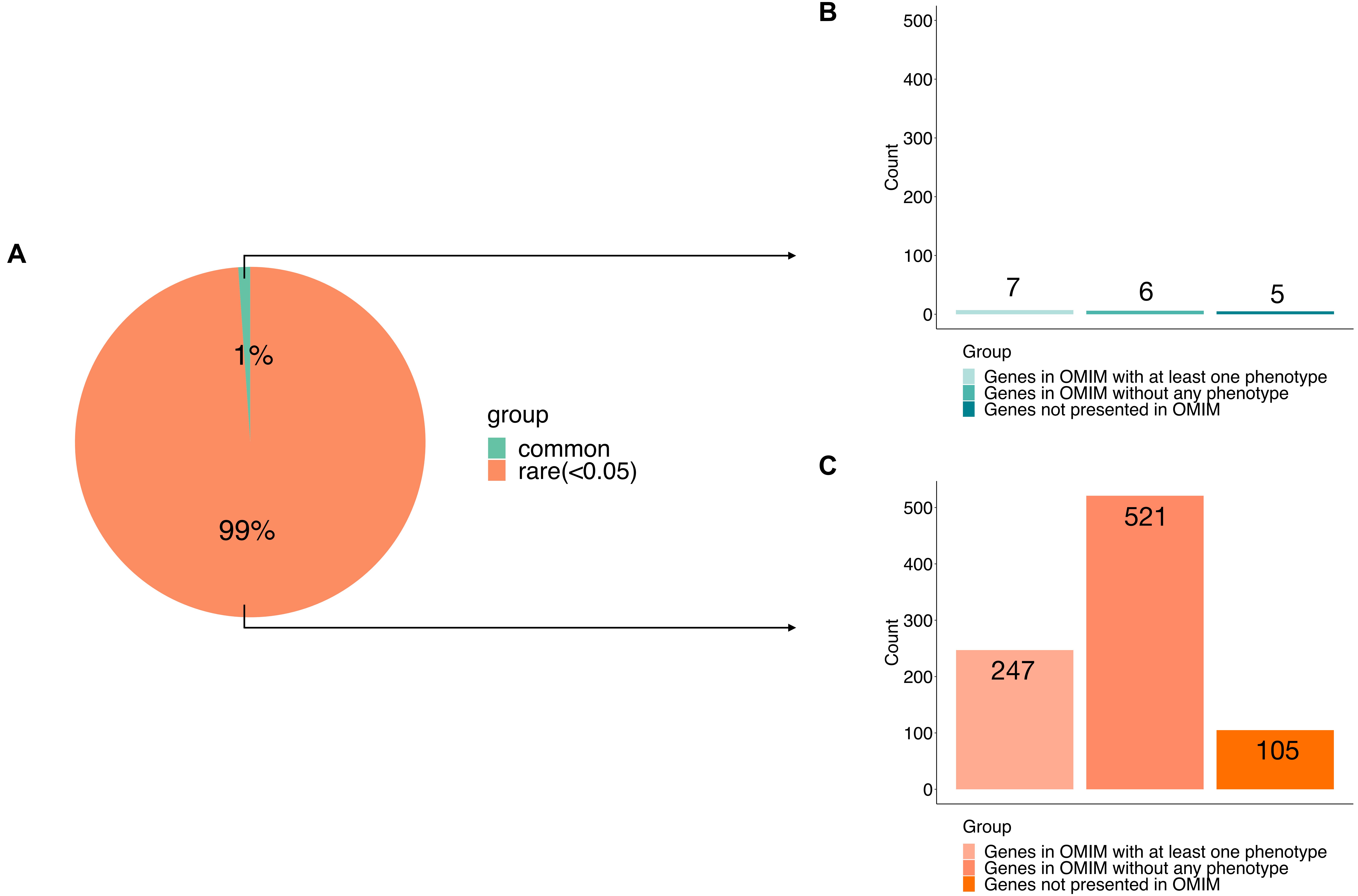

Supplementary figure 5

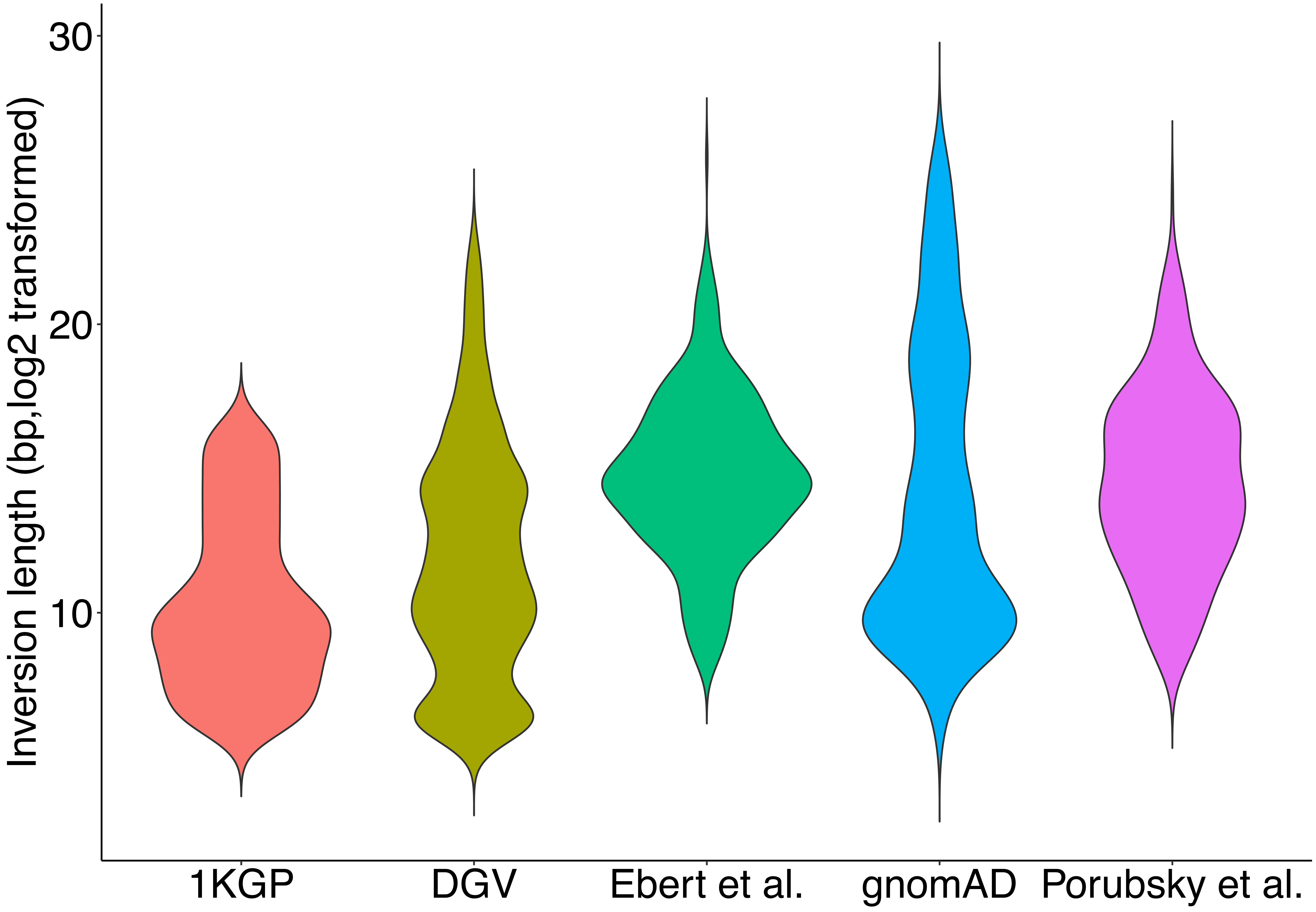

Supplementary figure 6

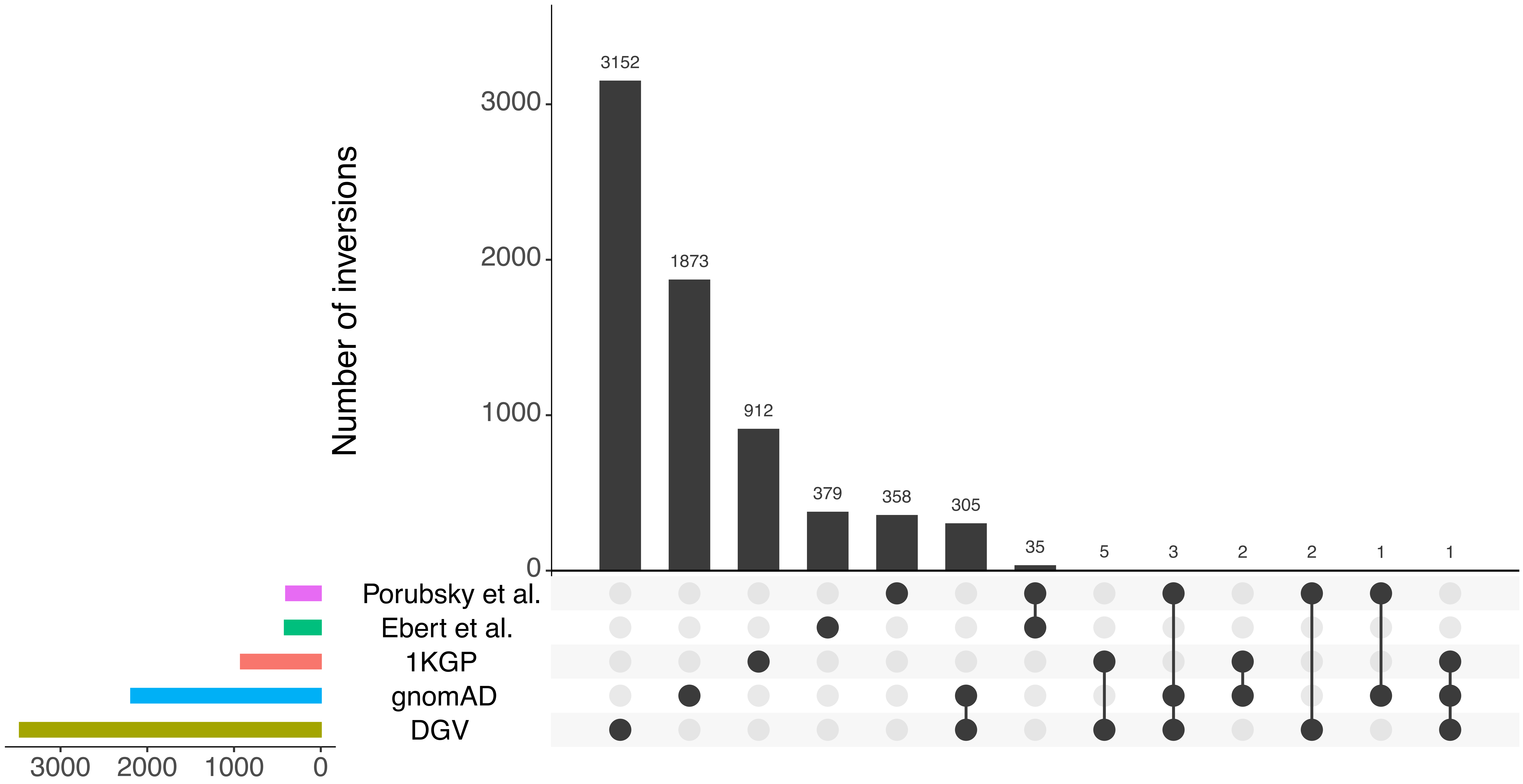

Supplementary figure 7

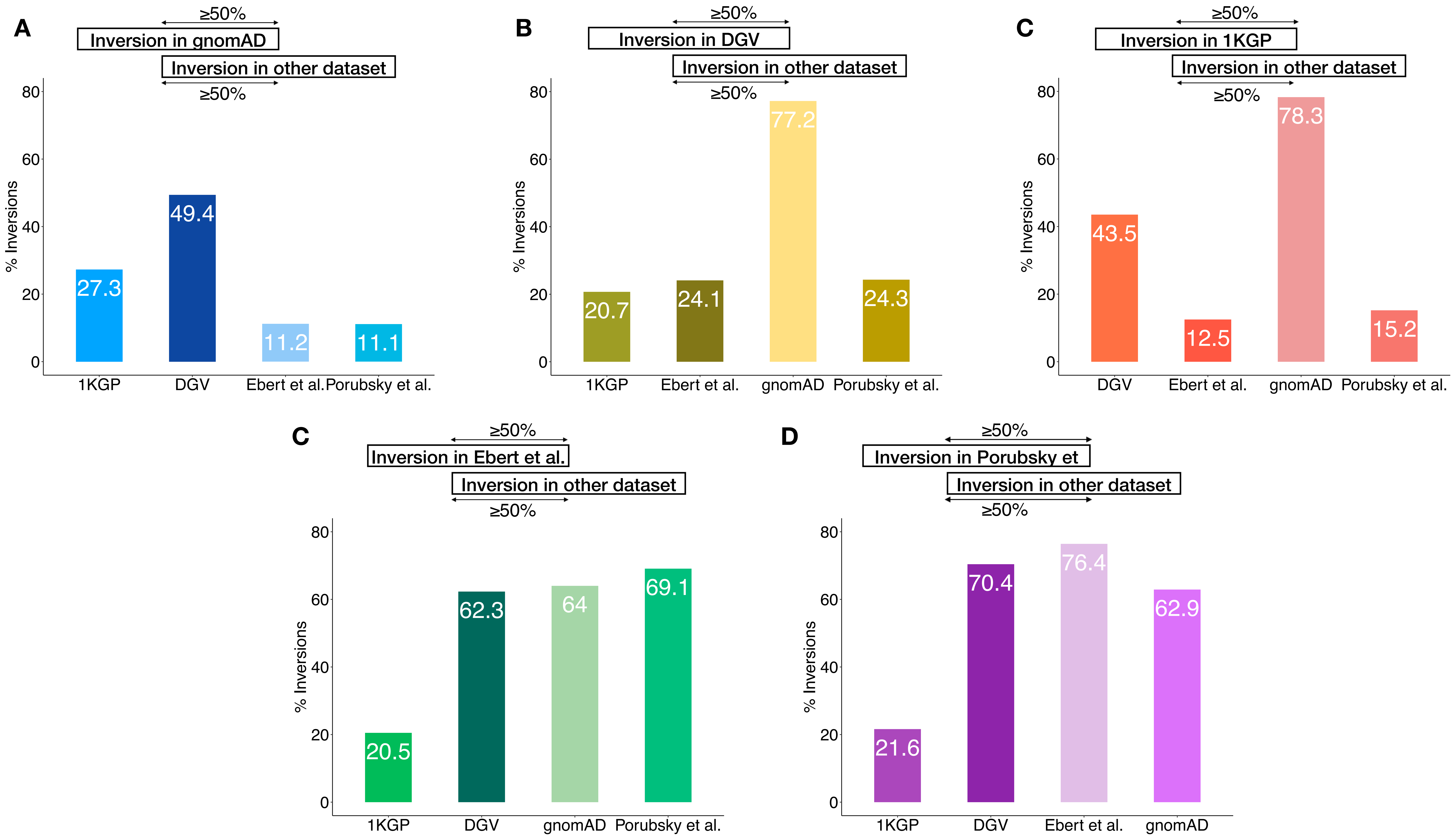

Supplementary figure 8

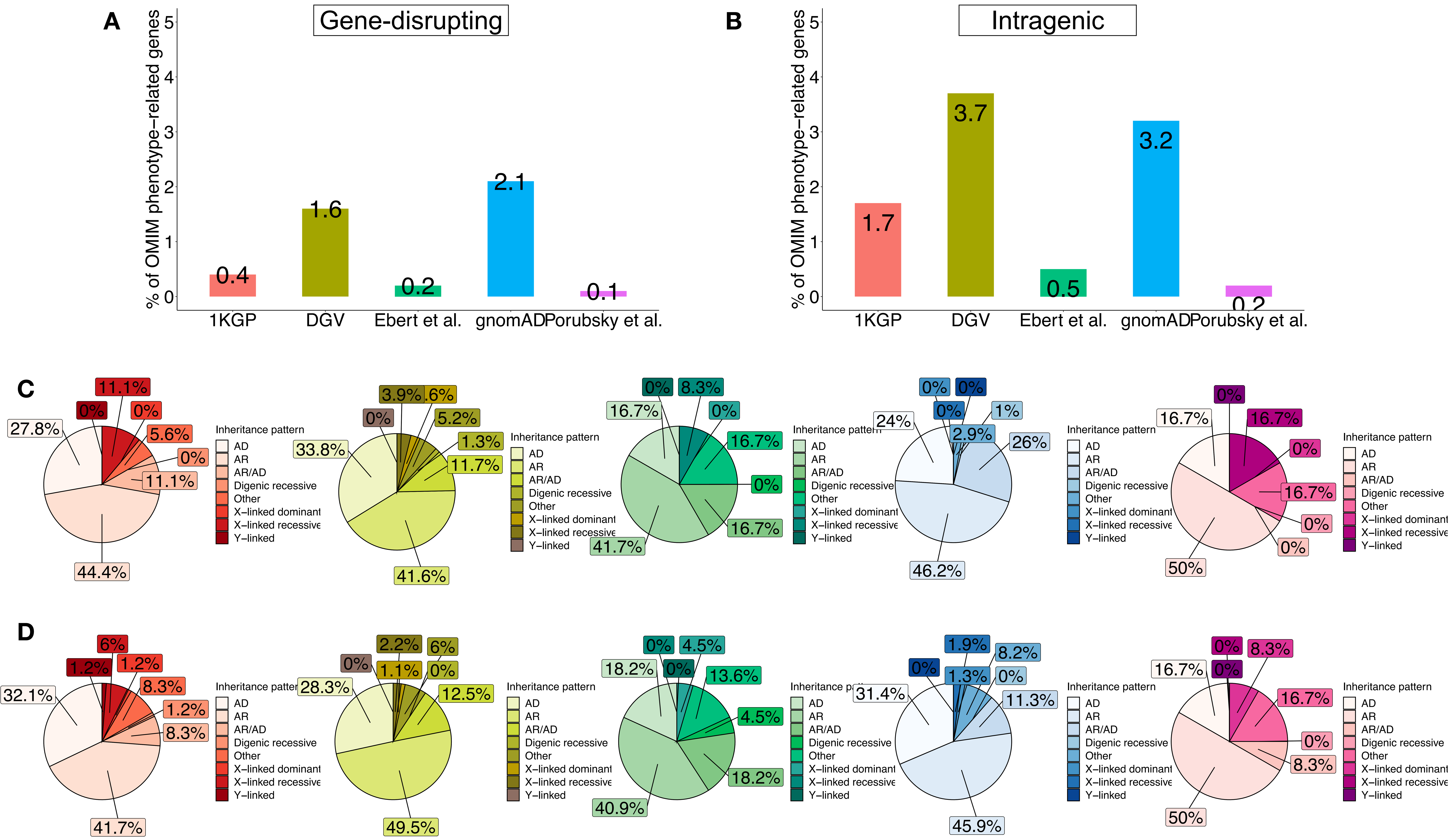

Supplementary figure 9

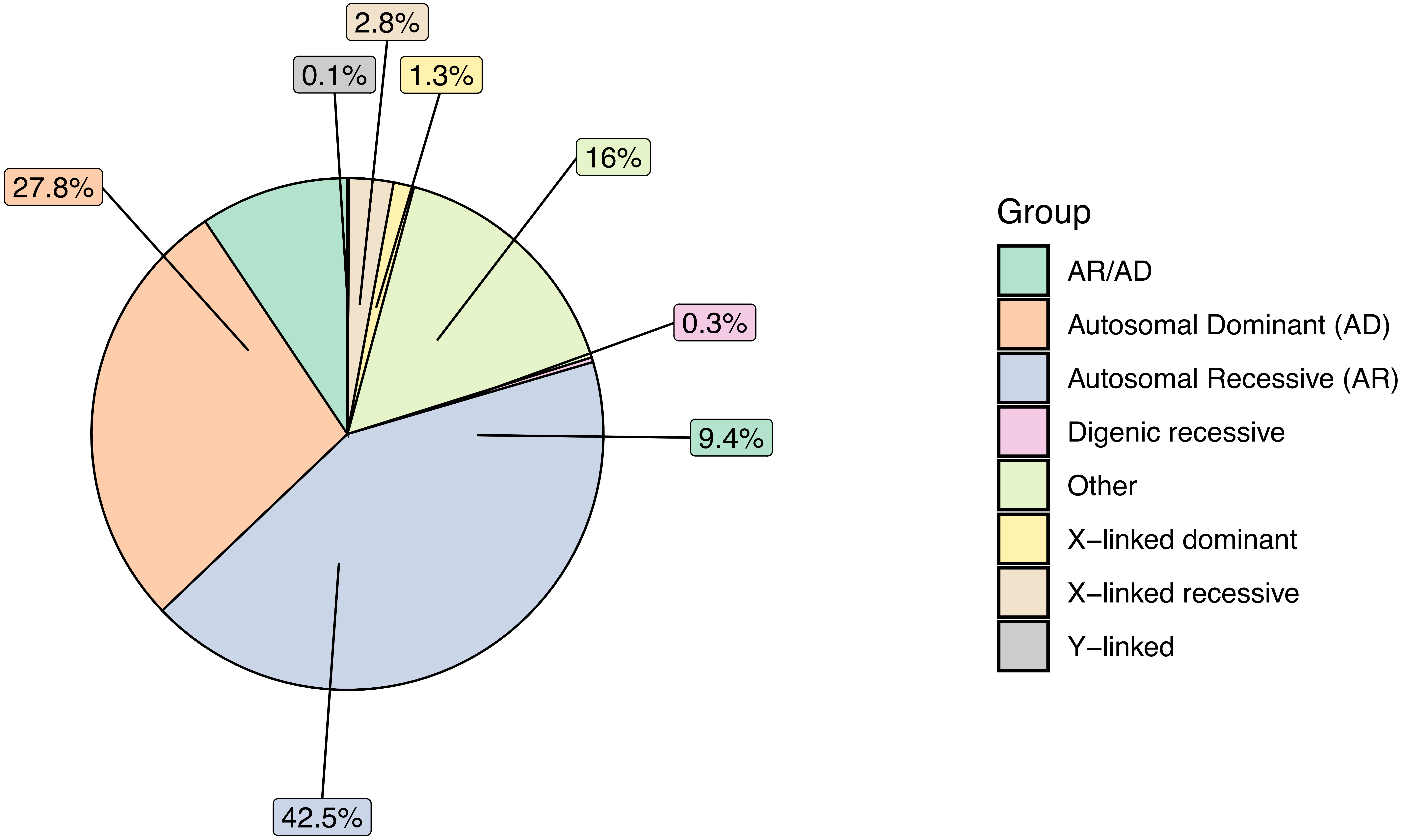

Supplementary figure 10
